# Impact on Sexual Function and Wish for Subsequent Pregnancy after Uterus-Preserving Prolapse Surgery in Premenopausal Women

**DOI:** 10.3390/jcm13144105

**Published:** 2024-07-13

**Authors:** Greta Lisa Carlin, Julia Hummel Jiménez, Sören Lange, Florian Heinzl, Marianne Koch, Wolfgang Umek, Barbara Bodner-Adler

**Affiliations:** 1 Department of Obstetrics and Gynecology, Medical University of Vienna, 1090 Vienna, Austria; 2 Department of Health Policy and Management, Harvard T.H. Chan School of Public Health, Boston, MA 02115, USA; 3 Department of Gynecology, University Hospital of Zurich, 8091 Zurich, Switzerland

**Keywords:** sexuality after prolapse operation, pregnancy after prolapse operation, sacrospinous hysteropexy, pregnancy after sacrospinous hysteropexy, sexuality after sacrospinous hysteropexy, pelvic organ prolapse

## Abstract

**(1) Background:** Pelvic organ prolapse (POP) affects millions of women globally, impacting their quality of life and potentially influencing family planning decisions. This study aimed to assess the impact of uterus-preserving prolapse surgery on the sexual function, desire for children, and pregnancy outcomes in premenopausal women with symptomatic POP. **(2) Methods:** A survey study was conducted among patients who underwent sacrospinous hysteropexy at a tertiary hospital between 2001 and 2021. Telephone interviews were performed to gather data on sexual function, desire for children, and satisfaction with surgical outcomes. **(3) Results:** The study included 33 premenopausal women, revealing diverse factors influencing sexual activity and desire for children following surgery. While most of the participants expressed a desire for children after surgery, sexually inactive individuals were more likely to report an unfulfilled desire for children. Fear of incontinence during sexual activity emerged as a significant concern for the sexually inactive participants. **(4) Conclusions:** The study highlights the need for comprehensive counselling and tailored interventions to address the multifaceted needs of women with POP. Further research is warranted to highlight the long-term implications of uterus-preserving surgeries on women’s health and well-being.

## 1. Introduction

Millions of women globally experience pelvic floor dysfunction, with some form of pelvic organ prolapse (POP) being observed in around 40% of women during examinations—although the self-reported and examination-based prevalences differ as POP can be asymptomatic in the early stages [1,2,3]. Although POP is generally more prevalent with older women, it can also affect younger women, with family history being a risk factor [4,5,6,7,8]. A 2014 US study found a self-reported symptomatic prevalence of POP of 1.6% in women aged 20–39 and 3.8% in women aged 40–59 [9]. While parity is generally associated with POP, notably [10], a Swedish national cohort study of nulliparae women under the age of 65 described the highest prevalence of symptomatic POP (9.8%) in the youngest age group (25–34 years) [11]. POP generally does not precipitate significant morbidity or mortality; nevertheless, it can detrimentally impact a woman’s sexuality, daily functioning, and overall quality of life [12] and thus potentially impact family planning as well. Furthermore, social taboos and shame still impede open scientific discourse [13].

While conservative management is usually the first step in the treatment of POP, surgical methods as treatment alternatives exist [2]. Moreover, the number of prolapse hysterectomies has declined significantly during recent years, and uterine-sparing alternatives have become more available [14]. These reconstructive native-tissue procedures have been described as the safest option for POP treatment in women wanting to preserve their fertility and wishing for future pregnancy [15]. Vaginal hysteropexy techniques, including the Manchester procedure, transvaginal uterosacral ligament suspension/plication, and sacrospinous hysteropexy, have been performed for many years [16]. Among these, sacrospinous hysteropexy (SSHP), attaching the uterus to the sacrospinous ligament [17], has emerged as a popular option, demonstrating satisfactory anatomic and functional outcomes [18]. 

Despite the fact that pelvic floor surgery is commonly performed in post-menopausal women [19], a significant proportion of patients opt to have these procedures during their reproductive years [20,21,22]. Furthermore, the recent trends indicate a rise in birth rates among older women [23], suggesting the possibility of pregnancy after pelvic floor surgery becoming more likely in the future [24].

Performing surgery for symptomatic POP in young women presents a complex multifaceted task, requiring the preservation of a patient’s fertility and sexual function while achieving satisfactory and long-lasting anatomical outcomes [25,26].

However, there is still little research and a lack of robust evidence regarding the impact of uterus-preserving surgeries, such as SSHP, on women’s sexual function and subsequent pregnancy. Therefore, we conducted a survey study to determine the sexual function, desire for children, and pregnancy outcomes in premenopausal women who underwent SSHP for symptomatic POP at our institution.

## 2. Materials and Methods

**Figure 1:**
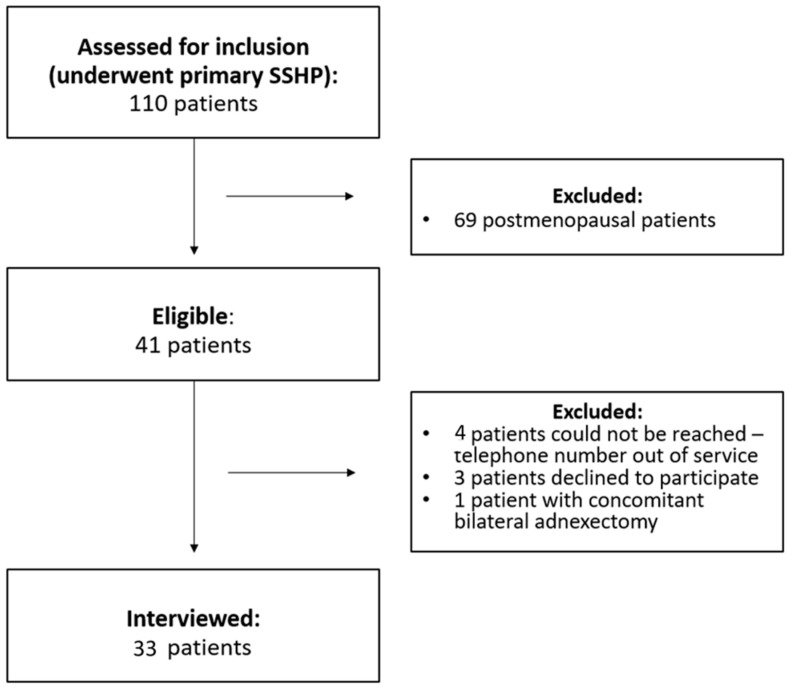
Flow chart of included patients.

A survey study was conducted; all patients who underwent primary SSHP for symptomatic POP (ICS POP-Q stage 2 or higher, Ref. [27]) between 2001 and 2021 at our tertiary hospital were eligible for inclusion. Demographic and clinical information as well as operative records were obtained from the patients’ electronic hospital charts, and written consent was obtained from all patients. All patient records were anonymized and de-identified prior to analysis. Of the 110 eligible patients, 41 women were premenopausal at the time of the surgery and included in the study. Inclusion criteria were all premenopausal patients undergoing a primary SSHP for symptomatic POP, with no history of pelvic or abdominal malignancy, and capable and willing to participate in the survey. Exclusion criteria included language difficulties, pelvic or abdominal malignancy, and prior prolapse hysterectomy. Telephone interviews were conducted between March and December 2023, and 33/41 eligible patients (80.5%) accepted to be interviewed by telephone (Figure 1).

### 2.1. Questionnaire

The telephone-based interviews consisted of the validated German questionnaire “PISQ-R Fragebogen” on sexuality after pelvic floor operations by Trutnovsky et al. [28]. This questionnaire is the translation of the validated Pelvic Organ Prolapse/Incontinence Sexual Questionnaire (PISQ-IR) [29]. In our survey, this questionnaire was further expanded by the researchers through following yes/no questions: “Do you have an unfulfilled desire to have children?”, “Did you postpone surgical POP treatment to fulfill a desire to have children?”, “Did you experience the desire to have children after SSHP surgery?”, “Did you experience fear at the thought of pregnancy after POP?”, and “Did you have a pregnancy after SSHP?”.

### 2.2. Statistical Analysis

The statistical analysis was performed using R version 4.2.2 (R Foundation for Statistical Computing). Descriptive statistics were calculated to summarize the demographic and clinical characteristics of the study participants. Continuous variables were presented as medians with interquartile ranges (IQRs), while categorical variables were presented as frequencies and percentages. To compare baseline characteristics and survey responses between sexually active and sexually inactive patients, we employed Mann–Whitney U Test (continuous variables), Chi-Squared Test (categorical variables), and Fisher’s Exact Test (categorical variables with small, expected frequencies). Statistical significance was set at *p* < 0.05. All analyses were conducted to evaluate trends and associations rather than to establish causal relationships given the observational nature of the study.

## 3. Results

### 3.1. Baseline Characteristics

The patients’ baseline characteristics are presented in Table 1, and no significant differences were observed between the sexually active and inactive patients following SSHP surgery. However, a significant association was found between body mass index (BMI) and sexual activity status (Table 1). The median BMI among those individuals who reported being sexually inactive was 29.37 (IQR: 26.23–32.59), whereas, among those who reported being sexually active, the median BMI was 24.3 (IQR: 21.88–27.9), suggesting a potential link between higher BMI and reduced sexual activity. 

### 3.2. Desire for Children

The majority of the participants, 28/33 (85%), expressed a desire to have children after surgery. Further, 8/8 (100%) of the sexually inactive women expressed a strong desire to have children compared to the sexually active individuals (20/25, 85%; *p* = 0.19). These results show a trend indicating that the sexually inactive individuals were more likely to express the desire to have children after SSHP compared to the sexually active individuals (Table 2).

A minority of the participants, 2/33 (6%), reported postponing surgical treatment for POP to fulfil their desire to have children, but no intergroup difference could be observed (*p* = 0.4729). Furthermore, the participants reported varying levels of fear of pregnancy after surgery, with the sexually inactive individuals showing a higher proportion of fear compared to the sexually active individuals (60% vs. 50%). However, this difference was not statistically significant either (*p* = 0.6875), and there was no significant difference in the occurrence of pregnancy after surgery between the sexually active and inactive groups (*p* = 0.8325) (Table 2).

### 3.3. Sexual Activity

The majority of the sexually inactive participants attributed their lack of sexual activity to reasons such as no interest (63%), other health problems (50%), and pain (38%). Nevertheless, a considerable proportion of the sexually inactive participants expressed dissatisfaction with their current sexual life (63%) and frustration due to pelvic floor dysfunction (38%). Fear of incontinence during sexual activity was a significant concern for the sexually inactive participants, with 63% reporting avoiding sexual activity due to this fear. Interestingly, most of the sexually inactive participants reported a neutral to positive impact of their partner on their sexual desire (91%) and sexual activity (95%; Table 3). 

The results of the PISQ-R questionnaire showed that most of the sexually active patients reported feeling sexually aroused weekly (56%), with their sexual desire rated as mid-range (72%). The majority of the sexually active patients reported feeling aroused during sexual activity (60%) and experiencing satisfaction (80%) and no shame (84%). The patients in this cohort also generally reported a positive influence of their partner on their sexual desire (91%) and sexual activity (95%). And, in contrast to the sexually inactive population, a significant proportion of the sexually active patients expressed satisfaction with their sexual life (76%) and minimal frustration due to pelvic floor dysfunction (24%).

## 4. Discussion

POP can negatively impact many aspects of an affected woman’s life, including sexual function. It could thus affect conception by increasing the issues already encountered when trying to conceive naturally and further delaying the process due to several reasons. POP can lead to a reluctance to engage in, limit the enjoyment of, or cause discomfort or even pain during penetrative intercourse. All these factors can create a barrier to conceiving.

Our study highlights the diverse factors contributing to sexual activity/inactivity and satisfaction following surgery, with a lack of interest, health problems, and pain being reported as significant factors contributing to sexual inactivity.

Fear of incontinence during sexual activity also emerged as a main concern for the sexually inactive participants in our study group. The influence of partners on sexual desire and activity varied; however, the partner’s impact was mainly positive, with some reporting a neutral impact and few negative effects. In regard to satisfaction, while a proportion of the sexually active patients reported feeling satisfied with their sexual life, many expressed frustration due to pelvic floor dysfunction—especially in the sexually inactive group. Furthermore, the frequency of sexual activity varied among the sexually active patients, with some reporting regular arousal and satisfaction, while others experienced lower sexual desire and satisfaction. It is further interesting to note that, although only premenopausal women were included in the study, eight out of the thirty-three patients reported not being sexually active at all.

Overall, the data show that pelvic floor dysfunction, incontinence, and other health-related factors significantly impact sexual activity and satisfaction. Additionally, the role of partners and individual perceptions of sexual health and function also play important roles in determining the sexual outcomes following surgery. All of these findings align with those of previously published studies [30,31].

Furthermore, while most of the women in our collective had already completed their family planning before considering a surgical treatment option for their symptomatic POP, 85.8% of the women reported to still have a desire to bear (further) children, and 6% expressed that they had delayed their POP treatment for fear of subsequent infertility; they were initially unaware of uterine-preserving operating techniques. It is thus essential to consider these findings in the context of the broader research on pelvic floor disorders and sexual health, as well as individual patient experiences and preferences.

In addition, despite most of the participants describing their family planning as complete before undergoing surgery, a significant proportion expressed a desire for further childbearing, underscoring the need for comprehensive counseling before surgery and the offer of tailored interventions.

The current literature recommends conservative therapy with a pessary for women who wish to bear children in the future as the first-line therapy [16,32]. However, additional surgical treatment options have to be evaluated for those women who do not tolerate pessary application. In addition, further research and tailored interventions are needed to address the multifaceted aspects of sexual well-being in premenopausal individuals with pelvic floor dysfunction.

As of now, there are no guidelines on how to counsel women with a symptomatic prolapse, a wish for surgery, and a desire for childbearing regarding uterus-sparing prolapse. The literature on the subject mainly consists of case reports and case series. A recently published retrospective case series describes 20 cases in which women gave birth after having had uterine-sparing POP surgery. The procedures included anterior and posterior colporrhaphy as well as apical suspension, and the majority of the women underwent cesarean delivery [33]. On the other hand, a Turkish retrospective study identified eight women who completed childbirth after SSHP and described SSHP as a suitable surgical option for women with symptomatic uterovaginal descensus who desire uterine preservation and future childbearing, and cesarean section as a dependable and satisfactory delivery method in these cases [34].

Barba et al. conducted a systematic review in 2021 including 151 patients who became pregnant after various prolapse surgeries [35]. Overall, adverse obstetrics events were rare (4.9%), and the authors came to the conclusion that native-tissue surgeries should currently be considered as the most cautious option.

Our findings contribute to the broader understanding of pelvic floor disorders and sexual health in premenopausal individuals, advocating for further research and personalized approaches to address these multifaceted issues.

### Strengths and Limitations

Our study was conducted at a single tertiary hospital. Its single-center, retrospective design limits the generalizability of the findings to broader populations. The survey was only conducted after the surgery, thus not enabling the comparison between pre- and postoperative findings. Reporter bias incurred through the nature of conducting the survey through telephone-based interviews can also not be fully excluded. However, the research group took care not to formulate leading questions and to note down each patient’s exact answers. Furthermore, only patients who underwent a primary SSHP for POP were included; therefore, no conclusions can be drawn regarding other uterine-preserving surgical management strategies for POP. Nevertheless, by reporting qualitative insights from telephone interviews, we can offer a comprehensive understanding of patients’ experiences and perspectives after SSHP for POP. It should be noted that, before 2015, SSHP was very rarely performed at our institution. Thus, the majority of the 110 eligible patients were operated on since then. However, efforts were undertaken to include all the possible patients to enhance the robustness of the findings, and inclusion was stopped in December 2021 to allow for a representative follow-up time before the survey.

## 5. Conclusions

In conclusion, this study provides valuable insights into the impact of uterus-preserving prolapse surgery on the sexual function, desire for children, and pregnancy outcomes in premenopausal women with POP. Most of the participants expressed a desire for bearing children, highlighting the importance of comprehensive counselling. Fear of incontinence during sexual activity emerged as a significant concern. While our study’s strengths include a substantial sample size and longitudinal follow-up, its single-center design and retrospective nature warrant further research. Multi-center prospective studies with longer-term follow-up are required to further elucidate the implications of uterus-preserving surgeries on women’s health and well-being. Nevertheless, our findings underscore the importance of personalized counseling and tailored interventions to address the multifaceted needs of women with pelvic floor disorders.

## Figures and Tables

**Table 1:** Patients’ baseline characteristics.

Variable		All	Sexually Inactive	Sexually Active	*p* Value
		33	8	25	
age (yrs.)	median (IQR)	45.36 (36.91–50.28)	48.25 (42.74–49.26)	44.99 (36.67–50.28)	0.5234
	[95% CI]	[40.23–48.93]	[30–52.03]	[37.32–49.83]	
BMI	median (IQR)	25.18 (22.9–28.82)	29.37 (26.23–32.59)	24.3 (21.88–27.9)	0.0125
	[95% CI]	[24–27.92]	[24–36.81]	[21.78–27.92]	
	NA	3 (9.09%)	0 (0%)	3 (12%)	
nicotine consumption	no	29 (87.88%)	7 (87.5%)	22 (88%)	0.9135
	yes	4 (12.12%)	1 (12.5%)	3 (12%)	
parity	median (IQR)	2 (2–3)	2.5 (1.75–3.25)	2 (2–3)	1.000
	[95% CI]	[2–3]	[0–6]	[2–3]	
POP-Q stage	median (IQR)	3 (2–3)	3 (2–3)	3 (2–3)	0.4940
	[95% CI]	[2–3]	[2–3]	[2–3]	
urinary incontinence	no	18 (54.55%)	4 (50%)	14 (56%)	0.5913
	yes	15 (45.45%)	4 (50%)	11 (44%)	
obstipation	no	25 (75.76%)	6 (75%)	19 (76%)	0.8413
	yes	8 (24.24%)	2 (25%)	6 (24%)	
fecal incontinence	no	30 (90.91%)	7 (87.5%)	23 (92%)	0.8229
	yes	3 (9.09%)	1 (12.5%)	2 (8%)	
happiness with SSHP outcome	no	3 (9.09%)	2 (25%)	1 (4%)	0.4888
	yes	30 (90.91%)	6 (75%)	24 (96%)	
recommendation of SSHP	no	5 (15.15%)	1 (12.5%)	4 (16%)	0.9856
	yes	28 (84.85%)	7 (87.5%)	21 (84%)	
time hospital -interview (days)	median (IQR)	1279 (801–2167)	872 (691.5–1770.25)	1309 (1050–2167)	0.1056
	[95% CI]	[983–1624]	[526–6178]	[1225–1913]	

**Table 2:** Family planning.

Variable		All	Sexually Inactive	Sexually Active	*p* Value
		33	8	25	
unfulfilled desire to have children	no	1 (33.33%)	1 (100%)	0 (0%)	0.250
	yes	2 (66.67%)	0 (0%)	2 (100%)	
	NA	30 (90.91%)	7 (87.5%)	23 (92%)	
desire to have children after SSHP	no	5 (15.15%)	0 (0%)	5 (20%)	0.2308
	yes	28 (84.85%)	8 (100%)	20 (80%)	
postponement of surgical POP treatment to fulfill desire to have children	no	31 (93.94%)	7 (87.5%)	24 (96%)	0.3723
	yes	2 (6.06%)	1 (12.5%)	1 (4%)	
fear of pregnancy after POP	no	3 (60%)	1 (100%)	2 (50%)	0.675
	yes	2 (40%)	0 (0%)	2 (50%)	
	NA	28 (84.85%)	7 (87.5%)	21 (84%)	
pregnancy after SSHP	no	30 (90.91%)	7 (87.5%)	23 (92%)	0.5716
	yes	3 (9.09%)	1 (12.5%)	2 (8%)	

**Table 3:** Results of PISQ-R questionnaire.

Sexually Inactive Participants				Sexually Active Participants			
Question		Answers	Patients	Question		Answers	Patients
			8				25
sexual inactivity caused by				how often do you feel aroused during sexual activity		rarely	1 (4%)
	lack of partner	fully agree	3 (37.5%)			sometimes	9 (36%)
		don’t quite agree	1 (12.5%)			usually	7 (28%)
		fully disagree	4 (50%)			as good as always	8 (32%)
	no interest	fully agree	5 (62.5%)	do you experience following during sexual activity:			
		don’t quite agree	1 (12.5%)		satisfaction	rarely	2 (8%)
		fully disagree	2 (25%)			sometimes	3 (12%)
	incontinence (urinary/fecal)	quite agree	3 (37.5%)			usually	11 (44%)
		don’t quite agree	1 (12.5%)			as good as always	9 (36%)
		fully disagree	4 (50%)		shame	never	15 (60%)
	other health problems	fully agree	4 (50%)			rarely	6 (24%)
		quite agree	2 (25%)			sometimes	2 (8%)
		fully disagree	2 (25%)			usually	2 (8%)
	pain	fully agree	3 (37.5%)		fear	never	17 (68%)
		don’t quite agree	1 (12.5%)			rarely	4 (16%)
		fully disagree	4 (50%)			sometimes	2 (8%)
avoiding sexual activity because of fear of incontinence		a little	2 (25%)			usually	1 (4%)
		some	1 (12.5%)			as good as always	1 (4%)
		a lot	5 (62.5%)	how often do you experience an incontinence episode during sexual activity		never	19 (76%)
agreement with following:						rarely	3 (12%)
	I am satisfied with my sexual life	fully agree	2 (25%)			sometimes	3 (12%)
		quite agree	1 (12.5%)	how intense are your sexual peaks in comparison to before		lower	3 (12%)
		agree some	2 (25%)			same	18 (72%)
		don’t quite agree	1 (12.5%)			higher	4 (16%)
		fully disagree	2 (25%)	how often do you experience pain during sexual activity		never	10 (40%)
	My sexual life suits my age	fully agree	5 (62.5%)			rarely	6 (24%)
		quite agree	1 (12.5%)			sometimes	7 (28%)
		don’t quite agree	1 (12.5%)			often	1 (4%)
		fully disagree	1 (12.5%)			always	1 (4%)
	My sexual life frustrates me	fully agree	3 (37.5%)	how often does your partner have a problem that impacts your sexual activity		often	1 (4.55%)
		quite agree	1 (12.5%)			sometimes	4 (18.18%)
		don’t quite agree	2 (25%)			seldom	17 (77.27%)
		fully disagree	2 (25%)			NA	3 (12%)
	I feel disadvantaged by my pelvic floor dysfunction	fully agree	1 (12.5%)	what is the impact of your partner on your sexual desire		very positive	11 (50%)
		don’t quite agree	2 (25%)			quite positive	9 (40.91%)
		fully disagree	5 (62.5%)			quite negative	2 (9.09%)
	I get angry because my pelvic floor disfunction impacts my sexuality	fully agree	1 (12.5%)			NA	3 (12%)
		don’t quite agree	2 (25%)	what is the impact of your partner on your sexual activity		very positive	11 (50%)
		fully disagree	5 (62.5%)			quite positive	10 (45.45%)
how bothered are you by your lack of sexual activity		not at all	3 (37.5%)			quite negative	1 (4.55%)
		a little	1 (12.5%)			NA	3 (12%)
		some	3 (37.5%)	do you feel as if you would like “more” when being sexually active		never	7 (28%)
		a lot	1 (12.5%)			rarely	7 (28%)
						sometimes	9 (36%)
						often	2 (8%)
				how often do you experience sexual desire		daily	3 (12%)
						weekly	14 (56%)
						monthly	4 (16%)
						less than once a month	3 (12%)
						never	1 (4%)
				how would you rate your sexual desire		high	5 (20%)
						mid-range	18 (72%)
						low	2 (8%)
				do you avoid sexual activity for fear of incontinence or POP sensation		not at all	15 (60%)
						a little	5 (20%)
						some	3 (12%)
						a lot	2 (8%)
				agreement with following statements:			
					I am satisfied with my sexual life	fully agree	12 (48%)
						quite agree	7 (28%)
						agree some	3 (12%)
						don’t quite agree	3 (12%)
					My sexual life suits my age	fully agree	13 (52%)
						quite agree	8 (32%)
						agree some	1 (4%)
						don’t quite agree	3 (12%)
					I feel secure and satisfied with my sexual life	fully agree	17 (68%)
						quite agree	5 (20%)
						agree some	1 (4%)
						fully disagree	2 (8%)
					My sexual life frustrates me	quite agree	2 (8%)
						agree some	4 (16%)
						don’t quite agree	19 (76%)
					I feel disadvantaged by my pelvic floor dysfunction	fully agree	2 (8%)
						quite agree	1 (4%)
						agree some	6 (24%)
						don’t quite agree	16 (64%)
					I am bothered by my sexual life	fully agree	1 (4%)
						quite agree	1 (4%)
						agree some	9 (36%)
						don’t quite agree	14 (56%)
					I get angry because my pelvic floor disfunction impacts my sexuality	fully agree	2 (8%)
						agree some	5 (20%)
						don’t quite agree	18 (72%)

## Data Availability

Individual participant data that underlie the results of this article after deidentification (text, tables, figures, and appendices) in addition to the study protocol and statistical analysis plan will be shared beginning 9 months and ending 36 months following article publication with researchers who provide a methodologically sound proposal. Proposal should be directed to the corresponding author. Data requestors will need to sign a data access agreement.
